# 530 Comparing Surgical Outcomes of Primary Closures to Split Thickness Skin Grafts in Patients with Burns

**DOI:** 10.1093/jbcr/iraf019.159

**Published:** 2025-04-01

**Authors:** Dat Tran, Heather Dodd, Lori Chrisco, Elisabeth Carter, Felicia Williams, Booker king

**Affiliations:** University of North Carolina at Chapel Hill; University of North Carolina Jaycee Burn Center; University of North Carolina Jaycee Burn Center; University of North Carolina; University of North Carolina at Chapel Hill; University of North Carolina

## Abstract

**Introduction:**

Primary closures (PC) and split thickness skin grafts (STSG) are two common methods to repair small deep partial or full thickness burns. We conducted an analysis comparing the surgical outcomes of PC and STSG containing average number of operations, percentage of a repeat operation, percentage of reported complications, average length of stay (LOS), average number of physical therapy (PT) and occupational therapy (OT) visits, average days of admit to healing, and average days of admit to compression.

**Methods:**

Patients were identified using the Burn Center registry and clinical data. All patients with a total body surface area (TBSA) burn ≤5% admitted between January 2019 and April 2024 who sustained a deep partial thickness or full thickness burn and had either a PC (n= 40) or STSG (n=91) were eligible for inclusion. The analysis evaluated several key metrics, including the number of operating room visits, reported complications including repeat operations, LOS, number of PT and OT visits, days of admit to healing, and days of admit to compression. The average number of operating room visits, LOS, number of PT and OT visits, days of admit to healing, and days of admit to compression were calculated using the T-Test Calculator for 2 Independent Means. Average LOS and number of PT and OT visits were calculated using Chi-Square.

**Results:**

A total of 131 patients were identified, 40 received PC and 91 received an STSG. Both groups were comparable in demographics, TBSA and number of operations. Average age of PC patients was 40.2 with 58% male, while the average age of STSG patients was 43.5 with 63% male. The average TBSA of PC patients was 2.5, while the average TBSA of STSG patients was 2.4. The average number of operations for both groups was 1.8. Contact burns were more likely to have a PC (30%) compared to a STSG intervention (12%). The percentage of patients who required a repeat operation was 25% for PC patients and 8.8% for STSG patients, p = 0.01 (significant). The percentage of reported complications was 17.5% for PC and 12.1% for STSG patients, p = 0.41. Average LOS was 13.8 days for PC patients and 11.6 for STSG patients, p = 0.48. Average number of PT visits was 6.7 visits for PC patients and 8.5 visits for STSG patients, p = 0.43. Average number of OT visits was 12.6 visits for PC patients and 9.7 visits for STSG patients, p = 0.30. Average days from admission to healed was 48.6 days for PC patients and 68.3 days for STSG patients, p = 0.12. Average days from admission to compression garments was 36.7 days for PC patients and 36.8 days for STSG patients, p = 0.98.

**Conclusions:**

PC patients experienced a higher average for a repeat operation compared to STSG patients. Based on this data, further research could be conducted to further look at the reasons why PC or STSG were chosen for a patient based on the composition of their burn wound and the risk factors each patient possesses.

**Applicability of Research to Practice:**

This study has the ability to help shape best clinical practice in patients with small burn injuries.

**Funding for the Study:**

N/A

